# Barriers and facilitators to taking on diabetes self-management tasks in pre-adolescent children with type 1 diabetes: a qualitative study

**DOI:** 10.1186/s12902-018-0302-y

**Published:** 2018-10-13

**Authors:** David Rankin, Jeni Harden, Katharine Barnard, Louise Bath, Kathryn Noyes, John Stephen, Julia Lawton

**Affiliations:** 1 The Usher Institute of Population Health Sciences and Informatics, University of Edinburgh, Edinburgh, EH8 9AG UK; 2 BHR Ltd, 42 Kilmiston Drive, Portchester, Fareham, Hants, PO16 8EG and Faculty of Health & Social Science, Bournemouth University, Royal London House, Bournemouth, BH1 3LT UK; 3 Royal Hospital for Sick Children, Sciennes Road, Edinburgh, EH9 1LF UK; 4 Child Health Department, Borders General Hospital, Melrose, TD6 9BS UK

**Keywords:** Type 1 diabetes, Children, Pre-adolescents, Self-management, Qualitative research

## Abstract

**Background:**

When children with type 1 diabetes approach adolescence, they are encouraged to become more involved in diabetes self-management. This study explored the challenges pre-adolescent children encounter when self-managing diabetes and the factors which motivate and enable them to take on new diabetes-related tasks. A key objective was to inform the support offered to pre-adolescent children.

**Methods:**

In-depth interviews using age-appropriate questioning with 24 children (aged 9–12 years) with type 1 diabetes. Data were analysed using an inductive, thematic approach.

**Results:**

Children reported several barriers to taking on self-management tasks. As well as seeking respite from managing diabetes, children described relying on their parents to: perform the complex maths involved in working out carbohydrate content in food; calculate insulin doses if they did not use a bolus advisor; and administer injections or insert a cannula in hard-to-reach locations. Children described being motivated to take on diabetes tasks in order to: minimise the pain experienced when others administered injections; alleviate the burden on their parents; and participate independently in activities with their peers. Several also discussed being motivated to take on diabetes-management responsibilities when they started secondary school. Children described being enabled to take on new responsibilities by using strategies which limited the need to perform complex maths. These included using labels on food packaging to determine carbohydrate contents, or choosing foods with carbohydrate values they could remember. Many children discussed using bolus advisors with pre-programmed ratios and entering carbohydrate on food labels or values provided by their parents to calculate insulin doses. Several also described using mobile phones to seek advice about carbohydrate contents in food.

**Conclusions:**

Our findings highlight several barriers which deter children from taking on diabetes self-management tasks, motivators which encourage them to take on new responsibilities, and strategies and technologies which enable them to become more autonomous. To limit the need to perform complex maths, children may benefit from using bolus advisors provided they receive regular review from healthcare professionals to determine and adjust pre-programmed insulin-to-carbohydrate ratios. Education and support should be age-specific to reflect children’s changing involvement in self-managing diabetes.

## Background

Type 1 diabetes (T1D) is one of the most prevalent chronic diseases among children [1], and its incidence is rising globally by 2–3% per year [2]. Ensuring children are involved in diabetes-related care from an early age is considered essential to promoting optimal glycaemic control and minimising risk of long-term complications [3–5]. However, the daily demands of managing T1D are complex and difficult, including the requirement to undergo procedures such as frequent daily blood glucose monitoring, injecting (around 4 times daily) or inserting insulin pump infusion sets (at least every 2–3 days); regulating food intake and counting carbohydrate; calculating insulin doses; and recognising and taking action to prevent or treat hypoglycaemia and hyperglycaemia [6]. For these reasons, diabetes management is considered too difficult for young children to do independently and parental involvement and supervision remain critical throughout childhood. As children move towards adolescence, they are encouraged to gradually assume more responsibility for diabetes-related tasks [7], ideally working in partnership with their parents [8], in order to establish their own self-management practices [9].

Limited research has explored factors and considerations which prompt pre-adolescent children to take on more diabetes-related responsibilities and how they might be best supported to do so. When qualitative research has been undertaken with children, studies have tended to include participants up to 18 years [10–13], or report a mix of children’s and parents’ views together [12, 14], which makes extrapolation of the findings to pre-adolescents more challenging. Previous qualitative research has also tended to focus on specific aspects of managing diabetes in childhood, including: knowledge of the role of insulin and risks of high/low blood glucose [11, 15]; accounts of sharing care with adults [11, 14–16]; emotional responses to living with diabetes [10–12, 17–19]; knowledge of self-care [11, 13, 15]; and, views about managing diabetes at school [20–22] and attending paediatric diabetes clinics [23–25].

To supplement and enhance this research, we conducted interviews with children aged 9–12 years with T1D. Our aim was to understand and explore the challenges children in this age group encounter self-managing diabetes and the factors and considerations which motivate and enable them to take on new self-management tasks. A key objective was to identify ways in which pre-adolescent children can be better supported to take on responsibility for self-managing diabetes. The decision to focus on children aged 9–12 years was made because pre-adolescence is a critical stage of transition between young childhood where children are dependent on their parents and the teenage years where they become increasingly independent and autonomous [14, 26].

## Methods

### Study design

We used in-depth interviews and age-appropriate questioning, which incorporated optional play-based tasks, to elicit children’s views. This design enabled each interview to be tailored to take into account differing ages and capabilities [27] and afforded the flexibility needed for children to discuss issues they perceived as salient, including those unanticipated at the study’s outset [28]. Data collection and analysis took place concurrently, enabling issues identified in early interviews to inform areas explored in later ones in line with an inductive approach.

### Recruitment and sample

Recruitment was undertaken in four Scottish paediatric diabetes centres located in diverse rural and urban catchment areas. After obtaining parents’ permission, health professionals approached children during routine clinical consultations using an opt-in procedure. Purposive sampling was used to ensure there was diversity in terms of children’s demographic and disease characteristics, and that approximately equal numbers of children using multiple daily injection (MDI) or pump regimens were recruited in line with usage by this age group across Scotland. To be eligible for the study, children needed to have been diagnosed at least 6 months to allow them to have had time to make emotional, physical and psychological adjustments to having T1D. Each participant, and their parents/carers, completed written, age-appropriate consent forms. Recruitment continued until data saturation was reached.

### Data collection

Interviews were conducted DR who had received professional training on ways to involve young children in research and the use of age-appropriate methods. Interviews were informed by a topic guide developed in light of literature reviews and revised in light of emerging findings. During their interviews, children were offered opportunities to use participatory activities, including drawing and game-playing tasks, to prompt discussion [29, 30]. Relevant areas explored during the interviews are shown in Table 1. Face-to-face interviews took place between July 2016 and February 2017 with one third of children choosing to have a parent/carer present. Interviews averaged 45 min, were digitally recorded and transcribed in full.

**Table 1:** Relevant areas explored in interview topic guides

• Children’s views, and experiences of, being involved in managing diabetes.	
• What do children recall about when and how they began to take on diabetes-related responsibilities; what were these tasks; and, why did they decide to become more involved?	
• Children’s perceptions of, and views about, the roles of significant others (e.g. parents, carers, family members, teachers, friends’ parents) in helping them to manage diabetes.	
• What are children’s views about taking on new responsibilities for managing diabetes; what tasks do they envisage undertaking; and, what are their reasons for wanting to have more involvement?	

### Data analysis

Interviews were analysed by DR and JL using a thematic approach informed by the method of constant comparison. Both researchers read all participants’ interviews in full before comparing them to identify issues and experiences which cut across different accounts [31]. Each researcher undertook their initial data analyses independently and wrote separate reports before meeting to discuss their interpretations and reach agreement on recurrent themes. A coding framework was then developed which captured key themes and contextual information needed to aid data interpretation. NVivo, a qualitative software package (QSR International, Doncaster, Australia), was used to facilitate data coding and retrieval and coded datasets were subjected to further in-depth analyses to identify sub-themes and illustrative quotations. Participants are referred to using unique identifiers throughout the paper.

Ethical approval was provided by the South East Scotland Research Ethics Committee 01, NHS Lothian (16-SS-0084).

## Results

The sample comprised 24 children (see Table 2). In keeping with findings of other studies [32–34], children’s involvement in diabetes related tasks, and the amount of support they received from their parents, was informed by their developmental maturity and individual needs. When invited to discuss their role and involvement in self-management, children highlighted various factors and considerations which had influenced their decisions to assume new responsibilities. All but one participant chose to take part in a conventional interview, hence our findings focus exclusively on quotations elicited during face-to-face conversations. Below, we begin by exploring the factors and considerations which hindered children from taking on new responsibilities, followed by the motivators and tipping points informing their decisions to become more involved, before concluding with factors which helped them transition to having a more independent role in managing their diabetes.

**Table 2:** Demographic characteristics of interview participants

Characteristic	N	%	Mean ± SD & range
Children (*n* = 24)			
Female	11	45.8	
Age – all children			10.3 ± 0.9, range 9–12
Female age at time of interview (Years)			10.4 ± 1.1, range 9–12
Male age at time of interview (Years)			10.2 ± 0.8, range 9–11
Female age at diagnosis (Years)			5.0 ± 3.0, range 1–10
Male age at diagnosis (Years)			6.7 ± 2.1, range 3–10
Diabetes duration – all children (Years since diagnosis)			4.3 ± 2.4, range 1–10
Regimen (at time of interview)			
Basal-Bolus	11	45.8	
CSII (Insulin pump)	13	54.2	

### Barriers to children taking on new self-management responsibilities

#### Over reliance on parents

Children reported how their parents undertook a range of diabetes management tasks on their behalf, including some which they described being competent to do themselves; in 015’s case, to allow her respite from managing diabetes when at home, or in 014’s, to allow her to have time to play:

“*sometimes I’m too lazy to do it. It means when I’m on the couch and my mum and dad tell me to do my finger [blood glucose check], then I’ll say, ‘no, you just do it’. It’s like mostly at night.” (015, aged 10)*.

“*at school I check myself and bolus. Em, but here [at home] I can’t be bothered bolusing [administering insulin] (laughter). So my mum or dad does it. And they do my [blood glucose] checks here too because I just want to play.” (014, aged 10)*.

Children of all ages also highlighted occasions when their parents undertook diabetes management tasks which they were normally capable of doing by themselves in order to protect them from harm. This included 005 who reported how her parents chose to administer insulin at times of day when they deemed she might be “too tired” to do so safely:

“*I don’t do the morning ones cause I’m too tired and I might make a mistake. So mum does my injections in the morning and dad does some of them in the evening… I kind of do about maybe 50% of my injections.” (005, aged 11)*.

#### Lacking mathematical skills: Bewilderment when counting carbohydrate and calculating insulin doses

Virtually all children described experiencing difficulties with diabetes management tasks which required them to perform complex maths. This included determining the carbohydrate contents of meals and snacks which, as 008 noted, left them reliant on adult caregivers:

“*there are some things that I just don’t know at all about. And I tend to just do what other people tell me because I always have a fear that I’m doing something wrong. So I do tend to let other people take control.” (008, aged 11)*.

A similar view was expressed by 012 who described knowing the carbohydrate value of specific items but being dependent on his father to work out carbohydrate in most foods, especially meals made from individual ingredients:

“*I know one or two things, like the things that are my favourite number [but] my dad always does the calculating. And I’m always sitting there. I’m just like- I’m waiting for him to tell me them. I’m there like, ‘What’s the carbs? What’s the carbs?’” (012, aged 9)*.

As many children also indicated, being unable to count carbohydrates meant they were sometimes unable to take part in activities with peers such as sleep-overs, going on school trips, or eating at friends’ houses because, as 004 explained:

“*[other] parents don’t know what to do like if I’m having, or going to like somebody’s for a barbecue, or something to eat, because they don’t know how to weigh it” (004, aged 10)*.

#### Difficulties calculating insulin doses without access to a bolus advisor

Further challenges were reported by individuals who described struggling to apply ratios and perform the maths needed to determine their insulin doses because, as 001 (aged 10) explained: “a lot of the diabetes stuff is like times it by four, then divide it by two and when it comes to division or fractions or decimals, I’m just not very good”. As 011, like others, discussed, this inability to perform complex maths, both to count carbohydrates and calculate insulin doses, meant he was dependent on his parents to undertake these tasks:

“*she’s [mother] really good at maths [and] she works out, like I think you work out everything about the jags [injections], and then I normally just turn it up to how many units, cause I hear her talk, like adding everything in her head, like she’s talking to herself. I’m like: wow (laughter) I don’t know what I’d do to learn that but it won’t be anytime soon.” (011, aged 10)*.

#### Accessing difficult to reach injection sites or locations to insert a cannula

Children also described having to rely on their parents to insert cannulas or administer injections when they were unable to reach certain parts of their bodies. This included several children using MDI regimens who had been advised to inject long-acting insulin into their buttocks to avoid over-using other sites which were used for meal-time injections. Similarly, several children who used pumps described depending on their parents to help insert a new cannula in difficult to reach locations:

“*because I do it round on my bum, so they [parents] need to hold the cannula down and I can press the button for it, for the needle to go in. And then they take it out.” (016, age 11)*.

### Motivations and tipping points to taking on self-management responsibilities

#### To minimise the pain experienced when injections were administered by others

Many children also reported motivations and tipping points which made them decide, or acknowledge that, it was an appropriate time to take on new self-management responsibilities. For example, several children who used MDI regimens, including 006, described choosing to administer their own injections because having other people perform this task was a source of discomfort:

“*I don’t like somebody pinching my skin and then doing my injections. So I like doing that to myself because I don’t feel very comfortable with people doing that. So my nurse in my school, she used to do that, and I needed to do it, cause I never felt very comfortable.” (006, aged 10)*.

A similar motivation was reported by 024, who described taking on responsibility for administering his own long-acting injections soon after being diagnosed when he was nine:

“*my dad stopped doing my night time insulin for me, because literally it was as sore as sore can be. … it’s usually that he put it in too quickly, that it makes like a really bad pain. So then I managed to do it by myself*.”

#### Alleviating parental burdens

Children also described taking on more self-management responsibilities to alleviate the burden on, and stress experienced by, their parents. This included 012 who described how, at age 9, he had decided to perform his own blood glucose checks and administer bolus doses of insulin using injections because:

“*[I’m] in school now, and it was like my mum and dad were getting really bothered having to come in every day. I decided like, well they’re getting annoyed at it, so I might as well just go ahead*.”

In other cases, children, including 013, described taking on new responsibilities if they noticed that their parents were struggling; for example, after beginning a new job or because of the pressures involved in caring for other dependents: “obviously she [mother] has to get dinner ready and all that, and see to everybody else. So it was just easier for her if like I knew how to do it [administer a bolus]” (013, aged 12). Similarly, 017 discussed wanting to learn how to change his pump infusion set in order to alleviate some of the demands his diabetes management placed on his mother when she was preparing other siblings for school:

“*I’d like to kind of know how to put in my cannula by myself to stop my mum getting stressed and all annoyed in the mornings because before school she tries not to be late. And she needs to change my cannula and stuff like that, get everybody dressed.” (017, aged 9)*.

#### Becoming more autonomous

As well as wanting to reduce demands on their parents, children described taking on self-management tasks so they could participate in activities with their peers. Several children, for example, discussed having learned how to calculate carbohydrates or change pump infusion sets so they could attend sleepovers at friends’ houses or spend nights away on school trips/camps or, in 004’s case, to be allowed to go and play at a friend’s house:

“*It’s only when I kind, like got to eight or nine that I started doing it [blood glucose checks] myself. We made a deal that if you [father] did my injections then I would do my bloods, so I needed to do it because if I was at my friend’s house, my dad wouldn’t be able to come and do it every time.” (004, aged 10)*.

#### Tipping points: Starting secondary school

Several older aged children (~ 11–12 years) also discussed how they would need to take on more self-management tasks when they transitioned to secondary school in order to adapt to having less dedicated support available from adults:

“*when I go to high [secondary] school, well I guess I’ll just have to be able to carb count by myself and do everything like that, because it’s much more different, cause you see we get a menu back for primary school. You get a menu and you pick… So you know what you’re having. So when you go to the academy [secondary school], you don’t know what’s on that day. So you go in and then you have to go line up at whatever queue you want for whatever you want.” (009, aged 11)*.

As well as recognising the need to become involved in counting carbohydrates, some children who used MDI regimens described how their forthcoming transition to secondary school had prompted them to consider using new locations on their bodies to administer injections because, as 022 described, he did not want to “miss out” on time spent with friends if he were to continue to remove clothing in order to inject:

“*[I’ll] probably learn how to do jags in more places, cause I can only do it in my arm myself … if you’re doing it in your arm you’ve got to take everything off. But with your belly you could slip, lift it up and do it under a table without going into another room.” (022, aged 10)*.

### Enablers to children taking on self-management responsibilities

#### Strategies to minimise needing to perform complex maths to count carbohydrates

As our findings have illustrated, some diabetes management tasks, such as counting carbohydrate contents in meals and determining insulin doses, were too difficult for children in this age group to do independently even if they were motivated to do so. To overcome these difficulties, some children described adopting strategies to limit the need to perform complex maths by choosing to eat snacks or meals with carbohydrate values they could remember:

“*If I’m having cereal in the morning and I have to work it out myself, I just say, ‘Mum, since I’m working it out today, can I please have toast?’ Or can you help me with the sub-division?” (001, aged 10)*.

In addition, children who used a pump which incorporated a bolus advisor, or those who used MDI regimens and had access to a combined bolus advisor and blood glucose monitor, described how using these devices helped them to calculate their own insulin doses because they did not have to perform complex maths using ratios: “I don’t have to do any working out and stuff, well you need to work out how many carbs, but the pump puts in the ratio for you” (003, aged 11). Some, including 008, also noted how, by using a bolus advisor, they did not need to take into account whether a correction dose was needed: “the pump does most of the work such as the correction, we don’t have to figure out how much insulin to put through for that. It just does it” (008, aged 11).

As several children further pointed out, by using labels from packets to calculate carbohydrates in meals/snacks, and having access to a bolus advisor, they were able to assume responsibility for calculating insulin doses in instances where they did not want their parents to be involved:

“*there’s like carbs on the food that I eat, like on the packet. So I just look at that and then I put that into my [blood glucose] monitor, and then I keep adding all of that up. And then, say 40 grammes add to 15, that would be like 55. Then I put that into my monitor and that would be possibly 6.5 or 6 units.” (006, aged 11)*.

While children struggled to count carbohydrates in meals made from multiple ingredients, several of those who used bolus advisors described how they were able to calculate their own insulin doses because their parents provided them with the total value of carbohydrate in their meals. For example, 018 (aged 11) described how for meals eaten at home, her mother “would tell me the grammes and my [blood glucose/bolus advisor] machine tells me how many units I’ve to get.” Similarly, children discussed the benefits of parents providing them with a note containing the carbohydrate values of individual food items or the total count in their school lunch:

“*my mum has like – she’s got it on a bit of paper. … And she sticks it on to my, well like my play piece [lunch box]. And then that’s how I know my carbs at school” (020, aged 9)*.

#### Mobile phones

In related accounts, children reported how mobile phone technology enabled them to self-manage diabetes without their parents being present. Specifically, children who used bolus advisors discussed using phones to contact their parents remotely when they needed advice about carbohydrate contents in order to calculate their own insulin dose: “if I’m away with my friends to say the cinema and like I don’t know the carbs of something, I’ll just text her [mother] or phone her and see if she knows” (010, aged 11). Similarly, others described using the camera on their phone to seek advice from parents about carbohydrate contents:

“*I have to take my phone everywhere, so I can take a picture, send it to her, and then she estimates how much [insulin] I put through for it. … I’m not too sure about when it comes to the technical level of figuring everything out” (008, aged 11)*.

## Discussion

This is one of the first qualitative studies to explore pre-adolescent children’s experiences of, and views about, taking on T1D self-management tasks. In keeping with findings from a study involving parents [14], children in our study highlighted various motivations and tipping points which prompted them to take on more diabetes-related responsibilities. These included: wanting to gain more autonomy and spend time away from parents, wishing to alleviate the burden diabetes management placed on parents, a desire to minimise discomfort arising from administering injections, and needing to make preparations to begin secondary school. However, our findings also illustrate several novel issues and challenges which children in this age group may encounter, principally those relating to difficulties experienced performing the complex maths needed to count carbohydrates and calculate insulin doses. Furthermore, children described how bolus advisor technology enabled them to assume more independent responsibilities for managing diabetes by limiting the need to perform complex maths and how mobile phones allowed them to seek advice about the carbohydrate content of meals when remote from parents.

A key finding in our study is that pre-adolescent children found it extremely difficult to perform the complex maths required to self-manage their diabetes. As others have shown, people with T1D require numeracy skills equivalent to a General Certificate of Secondary Education (GCSE) grade A-C in order to perform the complex maths involved in managing diabetes, including counting carbohydrates, taking into account physical activity, and using insulin-to-carbohydrate ratios [35]. While we might therefore expect pre-adolescent children to be numerically challenged because they have yet to receive comprehensive mathematical teaching in secondary school, the numerical complexities of diabetes management are not confined to pre-adolescents. Indeed, in line with our participants’ accounts, other studies have shown how adolescents and adults with T1D also encounter similar challenges [35–38], with poor numeracy skills being associated with lower levels of perceived self-efficacy and less participation in diabetes self-management behaviours [35, 36].

Reflecting findings from studies involving adults and parents of children with T1D diabetes [39, 40], our study has shown how having access to a bolus advisor allowed children to take on tasks which they hitherto found too challenging; specifically, by enabling them to calculate their own insulin doses without needing to use complex maths involving ratios. While a bolus advisor may be a useful and empowering tool for pre-adolescent children, physiological changes during childhood mean that a child’s insulin requirements can change very frequently [41], which requires corresponding adjustments to be made to pre-set carbohydrate-to-insulin ratios in bolus advisors. As our findings have shown, children are dependent on others to determine and pre-programme ratios into bolus advisors. While pre-adolescent children would not be expected to adjust pre-set ratios on their own, for bolus advisors to remain a useful and clinically appropriate tool, regular review by health care professionals should be undertaken to help ensure whether the correct ratio and basal rate settings are always being used.

We have also highlighted how children benefited from having access to a mobile phone with a camera because this enabled them to seek advice from their parents remotely about carbohydrates in meals eaten away from home. This use of mobile phone technology, however, inevitably resulted in a continued level of dependency on parents to supply information about carbohydrates. Hence, our findings suggest that children (and adults) with T1D might benefit from ongoing research to develop mobile phone applications capable of identifying in real-time the carbohydrate contents in meals [42].

Children also described how parents undertook diabetes management tasks on their behalf in order to provide them with respite and enable them to have a normal childhood. While these findings are reported in other studies involving children [19, 43], studies involving parents of children with T1D have also demonstrated that parents choose to undertake diabetes-related tasks such as administering injections, to alleviate the burden of self-management [16] and help preserve their child’s childhood [44].

Finally, our findings draw attention to how children’s involvement and motivations to self-manage diabetes can change as they move towards adolescence. Specifically, pre-adolescent children in our study described actively seeking ways to become more involved in managing diabetes so they could fit in with and spend time with their friends and because they anticipated that less support would be available when they transitioned to secondary school. However, studies involving adolescents with T1D have demonstrated that the same motivations, particularly a desire to fit in with peers, can result in individuals in this older age group compromising their treatment regimens, including skipping blood glucose checks or administering insulin to avoid interrupting social activities [10, 43, 45, 46]. When taken together, findings from our own and adolescent studies indicate that a uniform approach to diabetes education is unlikely to address the needs of children of differing ages. Hence, we would recommend that education and support programmes should be age-specific and take into account children’s changing involvement in diabetes-related tasks.

A key strength of our study is our use of an open-ended exploratory design using age-appropriate questions, as this has enabled us to identify a number of potentially important issues which have not yet been recognised or reported in the literature. An additional strength is related to the timing of our study as this enabled us to highlight and explore how new technologies, such as bolus advisors, can enable pre-adolescent children to assume more diabetes-related responsibilities. A potential limitation is that we had a mostly White, ethnically homogenous sample, which potentially limits the generalisability of the findings. Our sample size also limits exploration of how individual factors such as diabetes duration, age at diagnosis and pubertal status might affect pre-adolescent children’s self-management decisions and future researchers might consider using quantitative methods to investigate these areas more fully. While the timing of our study enabled us to explore children’s use of technology such as bolus advisors, technological advances will result in further changes to how children are involved in self-managing diabetes. Hence, we recommend that future studies exploring the development of self-management roles should include children from more ethnically diverse groups and those who use newly emerging technologies, such as such as continuous glucose monitoring and/or closed loop systems [47], and flash glucose monitoring [48].

## Conclusions

This is one of the first qualitative studies to explore in depth factors and considerations which affect pre-adolescent children’s decisions to take on responsibilities for self-managing T1D. Our findings identify several factors which may hinder children from taking on self-management tasks, motivators and tipping points which influence whether they take on new responsibilities, and how new technologies can help them to become more independent. To address the numerical challenges involved in managing diabetes, children may benefit from using bolus advisors which limit the need to perform complex maths, alongside regular review from health care professionals to adjust and re-programme insulin-to-carbohydrate ratios. Children (and parents) may also benefit from education and support which is age-specific to reflect their changing involvement in diabetes-related tasks.

## Abbreviations

T1D: Type 1 diabetes
